# Acclimation of circadian rhythms in woodland strawberries (*Fragaria vesca* L.) to Arctic and mid-latitude photoperiods

**DOI:** 10.1186/s12870-023-04491-6

**Published:** 2023-10-10

**Authors:** Corine Faehn, Michael Reichelt, Axel Mithöfer, Timo Hytönen, Jørgen Mølmann, Laura Jaakola

**Affiliations:** 1 Department of Arctic and Marine Biology, The Arctic University of Norway, Tromsø, 9037 Norway; 2 Department of Biochemistry, Max Planck Institute for Chemical Ecology, 07745 Jena, Germany; 3 Research Group Plant Defense Physiology, Max Planck Institute for Chemical Ecology, 07745 Jena, Germany; 4 Department of Agricultural Sciences, Viikki Plant Science Centre, University of Helsinki, Helsinki, 00790 Finland; 5 NIBIO, Norwegian Institute of Bioeconomy Research, P.O. Box 115, Ås, 1431 Norway

**Keywords:** Arctic, Mid-latitude, Photoperiod, Daylength, Circadian clock, Phytohormones, Circadian rhythm, *Fragaria vesca*, Plant adaptation

## Abstract

**Background:**

Though many abiotic factors are constantly changing, the photoperiod is a predictable factor that enables plants to time many physiological responses. This timing is regulated by the circadian clock, yet little is known about how the clock adapts to the differences in photoperiod between mid-latitudes and high latitudes. The primary objective of this study was to compare how clock gene expression is modified in four woodland strawberry (*Fragaria vesca* L.) accessions originating from two different populations in Italy (IT1: Tenno, Italy, 45°N, IT4: Salorno, Italy, 46°N) and two in Northern Norway (NOR2: Alta, Norway, 69°N, NOR13: Indre Nordnes, Norway 69°N) when grown under simulated daylength conditions of an Arctic or mid-latitude photoperiod. The second objective was to investigate whether population origin or the difference in photoperiod influenced phytohormone accumulation.

**Results:**

The Arctic photoperiod induced lower expression in IT4 and NOR13 for six clock genes (*FvLHY, FvRVE8, FvPRR9, FvPRR7, FvPRR5*, and *FvLUX)*, in IT1 for three genes (*FvLHY, FvPRR9*, and *FvPRR5*) and in NOR2 for one gene *(FvPRR9*). Free-running rhythms for *FvLHY* in IT1 and IT4 were higher after the Arctic photoperiod, while the free-running rhythm for *FvLUX* in IT4 was higher after the mid-latitude photoperiod. IT1 showed significantly higher expression of *FvLHY* and *FvPRR9* than all other accessions, as well as significantly higher expression of the circadian regulated phytohormone, abscisic acid (ABA), but low levels of salicylic acid (SA). NOR13 had significantly higher expression of *FvRVE8, FvTOC1*, and *FvLUX* than all other accessions. NOR2 had extremely low levels of auxin (IAA) and high levels of the jasmonate catabolite, hydroxyjasmonic acid (OH-JA).

**Conclusions:**

Our study shows that circadian rhythms in *Fragaria vesca* are driven by both the experienced photoperiod and genetic factors, while phytohormone levels are primarily determined by specific accessions’ genetic factors rather than the experienced photoperiod.

**Supplementary Information:**

The online version contains supplementary material available at 10.1186/s12870-023-04491-6.

## Background

The photoperiod (daylength) is a highly predictable factor that has prompted one of the most evolutionarily conserved time-keeping mechanisms in all domains of life on Earth, a circadian clock. Mid-latitudes experience moderate seasonal variations in photoperiod compared to the equator, varying from approximately 9–15 h between winter and summer. Whereas the annual changes in photoperiod in the polar regions are extreme, ranging from nearly 24 h of photosynthetic light during summer (midnight sun) to no photosynthetic light during winter. This restricts the growth season to spring and summer due to the rapid shortening in photoperiod in autumn, which affects the timing of plant growth, flowering, biomass production, and quality [1]. The circadian clock is an important factor in physiological adaptations across regional scales [2], but how the clock balances between stability and plasticity across middle and high latitudes is unknown. The woodland strawberry, *Fragaria vesca*, is an herbaceous perennial with a wide geographic distribution from 37°N-70°N across the northern hemisphere. With a small, fully sequenced genome (*2n = 14, ~ 200 Mb*), and shared sequence identity with the cultivated octoploid strawberry (*Fragaria* x *ananassa, 2n = 56, ~ 813 Mb*), this plant is an ideal model to study clock plasticity to photoperiodic differences [3–5].

**Fig. 1:**
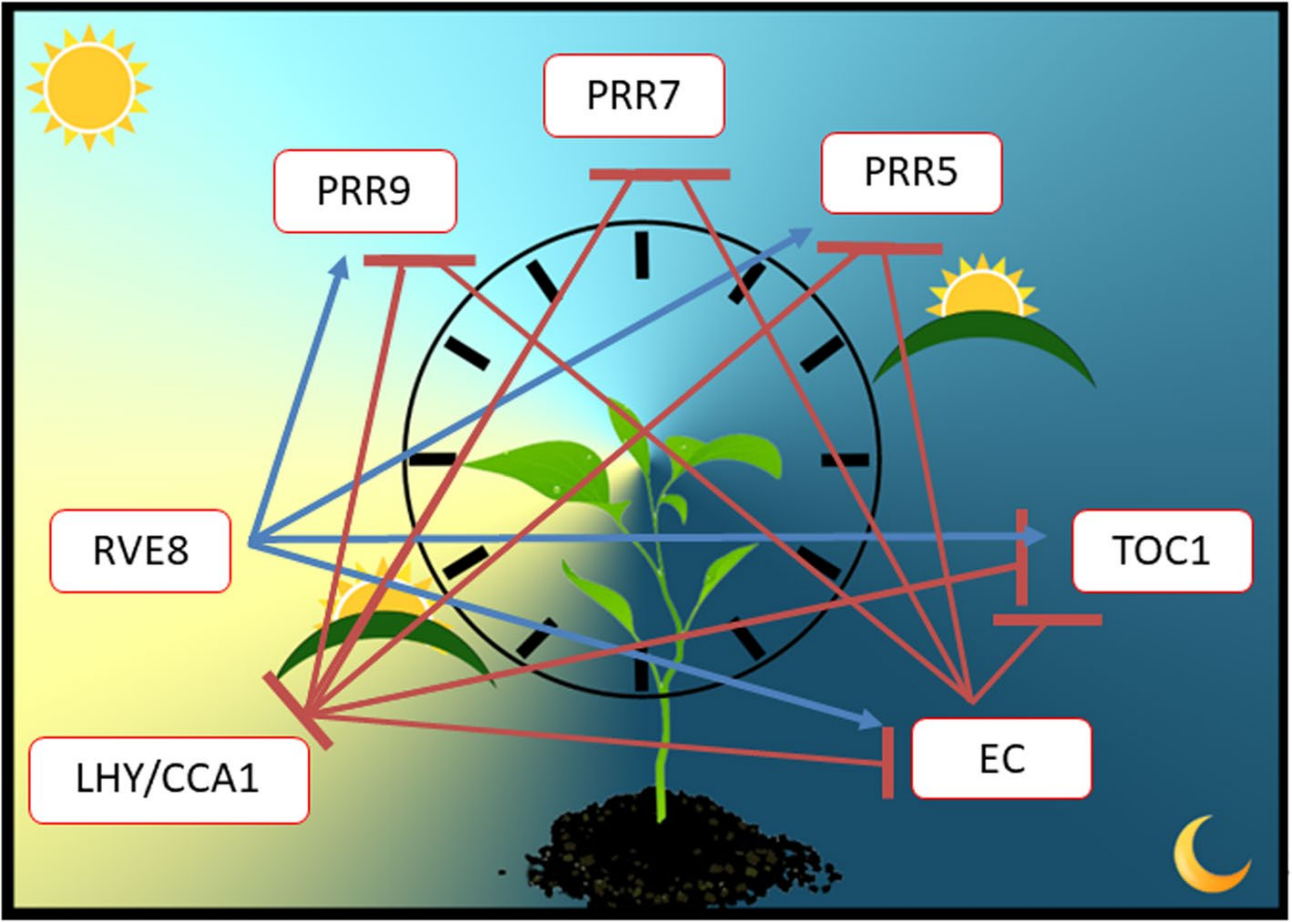
The core plant circadian clock. A simplified model of the core plant circadian clock which connects the morning, daytime, and evening-phased genes in a three-component repressor/activator system which acts: (1) *LHY/CCA1* repress the Evening Complex (EC: *LUX, ELF3*, and *ELF4*) while *RVE8* activates the EC; (2) the EC represses *PRR* genes (*PRR9, PRR7, PRR5*, and *TOC1*); and (3) *PRR* genes repress *LHY/CCA1*.

The mechanism of the circadian clock in plants has the same transcription-translation feedback loop (TTFL) found in almost all other organisms, comprised of a core set of genes which function as transcriptional activators [6] and/or repressors [7–10] of other clock components (Fig. 1). Although *Arabidopsis* has received considerable attention in the study of functional characteristics of clock components in plants, it is important to recognize that the function of individual components can vary among different species [11]. Therefore, it is imperative to evaluate the plasticity of the clock in other species to understand how the clock system can adapt to new regions under future climate scenarios.

In most plants, two morning-expressed genes, *CIRCADIAN CLOCK ASSOCIATED 1* (*CCA1*), and/or *LATE ELONGATED HYPOCOTYL* (*LHY*), are essential components to the core clock. Not all plants contain both *CCA1* and *LHY* genes, and *F. vesca* contains only *LHY* [12]. CCA1 and LHY proteins function as transcriptional repressors of their own transcription as well as many other clock genes [9, 13]. As CCA1 and LHY levels decline throughout the day, daytime and evening phased genes are then transcribed which further represses transcription of *CCA1* and *LHY* [7, 9]. *CCA1* and *LHY* are two partially redundant MYB-domain containing transcription factors that belong to the small REVEILLE subfamily including the close homolog *REVEILLE 8* (*RVE8*). *RVE8* is the main transcriptional activator of evening genes by targeting an evening element (EE) motif in their promoter regions [6].

Daytime expressed genes play roles within the regulation of both morning and evening-phased complexes of the clock. Members of the *PSEUDO-RESPONSE-REGULATOR* gene family (*PRR9, PRR7*, and *PRR5*) are repressed by *LHY* and are said to express sequentially throughout the day in *Arabidopsis* [13, 14], however, in *F. vesca PRR9, PRR7, and PRR5* have been shown to express at nearly the same time around noon [12]. These gene function as transcriptional repressors of the morning genes *CCA1, LHY*, and *RVE8* [15]. An evening-expressed *PRR*, *TIMING OF CHLOROPHYLL A/B BINDING PROTEIN 1* (*TOC1* or *PRR1*), is an indispensable gene to the core clock network, which functions to repress expression of morning-expressed genes at night [16]. An Evening Complex (EC) composed of *LUX ARRYTHMO* (*LUX*) also known as *PHYTOCLOCK 1* (*PCL1*), and *EARLY FLOWERING 3* & *4* (*ELF3* & *ELF4*) downregulates transcription of *TOC1* after dusk, allowing transcription of *CCA1* and *LHY* to restart the next dawn [13, 17]. In *F. vesca, TOC1* and *LUX* were the only evening genes that displayed circadian rhythms [12]. Thus, the interplay of activators and repressors keeps the clock components expressed during the right phase of the day.

The key function of the core genes involved in the plant clock also tightly regulate many output pathways. More than 1/3 of the transcriptome in plants is regulated by the circadian clock [18]. Mechanisms that enhance tolerance to UV radiation or drought can provide increased resistance to abiotic stressors, while mechanisms that enhance the timing of gene-mediated defenses or elevate levels of protective hormones can boost immune responses during periods when pathogens are expected to be active [19, 20]. For example, LHY interacts with the biosynthetic pathways of abscisic acid (ABA), which function under drought and osmotic stress [21], while regulation of jasmonic acid (JA) [22], and indole-3-acetic acid or auxin (IAA) [23] are regulated by multiple levels of the circadian network to increase tolerance to biotic stress as well as regulate plant growth and development. It has been hypothesized that jasmonate biosynthesis is promoted by high-latitude summer photoperiods which increases constitutive defense against herbivory [24].

It has been shown that plants at higher latitudes have longer circadian periods of leaf movement [25, 26], however, how these very long photoperiods affect the molecular plant circadian clock, and its downstream regulation, is much less known. Here we study the effect of differences in photoperiod between Arctic latitudes and mid-latitudes on the woodland strawberry circadian clock. To do this, two *F. vesca* accessions from Italy (IT1: Tenno, Italy, 45°N, IT4: Salorno, Italy, 46°N) and two from Northern Norway (NOR2: Alta, Norway, 69°N, NOR13: Indre Nordnes, Norway, 69°N), were grown under simulated daylength conditions associated with the growth season at an Arctic and mid-latitude photoperiod. First, the expression patterns of seven core clock genes were analyzed, followed by analysis of phytohormone accumulation. This study furthers the understanding of how plants adapt their circadian rhythms to new photoperiods.

## Results

### Plant biomass grown under Arctic and mid-latitude photoperiods

One accession (NOR13) had significantly lower biomass under the Arctic photoperiod (0.62 ± 0.15 g, 0.39 ± 0.13 g, *p* = 0.04). All other accessions were not significantly affected by photoperiod (IT1: 1.02 ± 0.14 g, 0.96 ± 0.23 g, IT4: 0.62 ± 0.20 g, 0.62 ± 0.23 g, NOR2: 0.64 ± 0.22 g, 0.84 ± 0.37 g).

### Differences in circadian clock gene expression between Arctic and mid-latitude photoperiods and the effect on free-running rhythms

Six of the seven clock genes (all but *FvTOC1*) had distinct phases of expression. Here, ZT 24 is the same point of the light cycle as ZT 0, however it is not grouped with ZT 0 for statistical comparison for free-running rhythms. All post-hoc results are listed in Suppl. Table 2 for significant differences between the treatments, and Suppl. Table 3 for significant differences between the accessions.

**Fig. 2:**
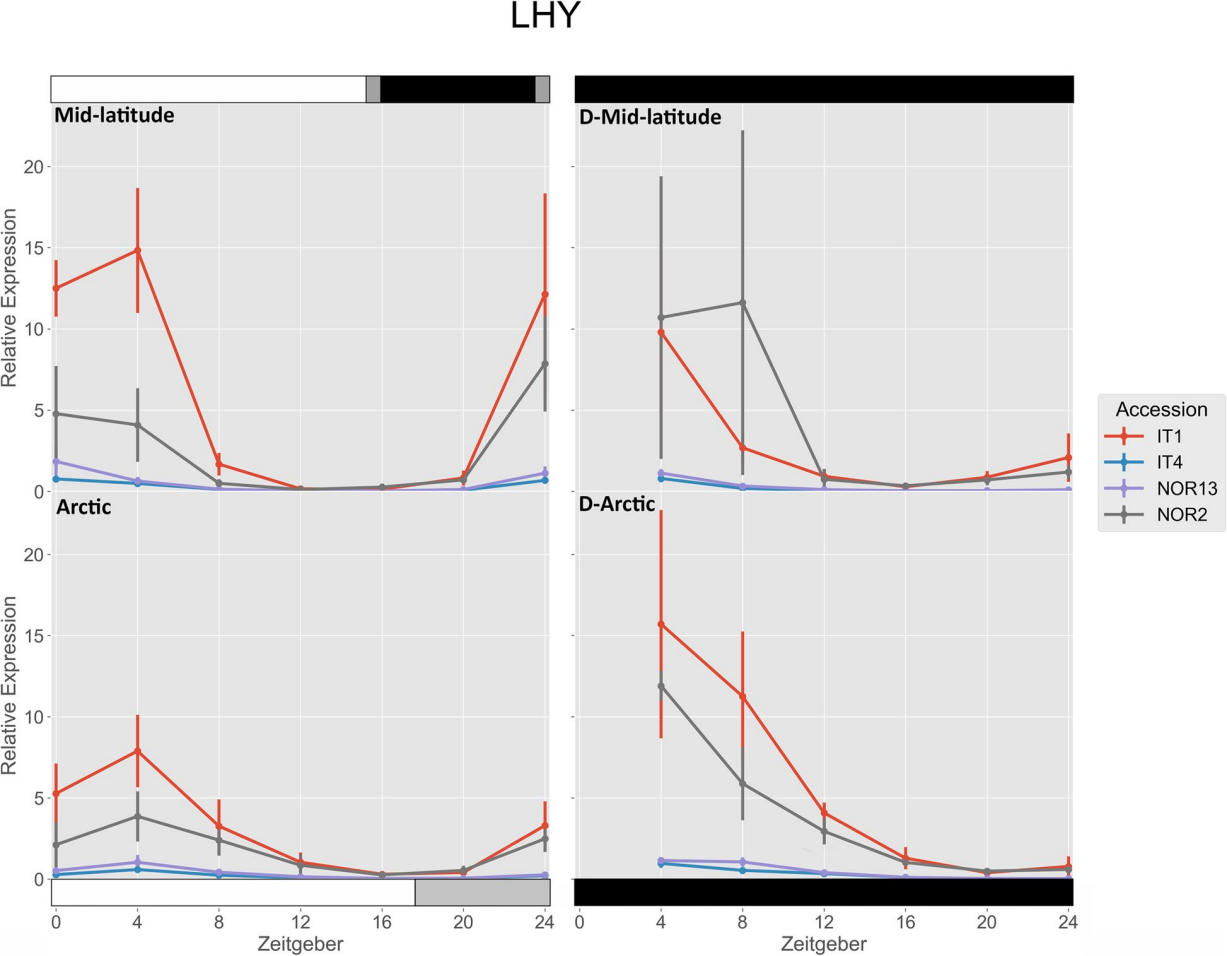
Relative expression of *FvLHY*. The photoperiod conditions are indicated by the bars on the top and bottom, where the white bars indicate photosynthetic light, gray bars indicate non-photosynthetic light, and black bars indicate total darkness. ‘D-‘ represents when the plants were under total darkness for free-running rhythms. Each point for the light treatments represents the mean ± std of the 2^-ΔCq^ values of three biological replicates over two consecutive 24 hour periods, and each point for the free-running rhythms is over one 24 hour period

IT1, IT4, and NOR13 had significantly higher expression of *FvLHY* under the mid-latitude photoperiod compared to the Arctic photoperiod at ZT 0/24 (Fig. 2). Under the mid-latitude photoperiod, IT1 had significantly higher expression than all other accessions at ZT 0, and ZT 4, while NOR2 was significantly higher than IT4 at ZT 0 and ZT 4. Under the Arctic photoperiod, IT1 had significantly higher expression than all other accessions at ZT 0 and ZT 4, while NOR2 was significantly higher than IT4 and NOR13 at ZT 4 and ZT 8. Free-running rhythms after the mid-latitude photoperiod were significantly higher for IT4 at ZT 4, and NOR2 at ZT 8, but significantly lower for IT1, IT4, and NOR13 at ZT 24. Free-running rhythms after the Arctic photoperiod were significantly higher for IT1 and IT4 at ZT 4 and 8. The free-running rhythms were also significantly higher for IT1 and IT4 at ZT 8 after the Arctic photoperiod compared to after the mid-latitude photoperiod.

**Fig. 3:**
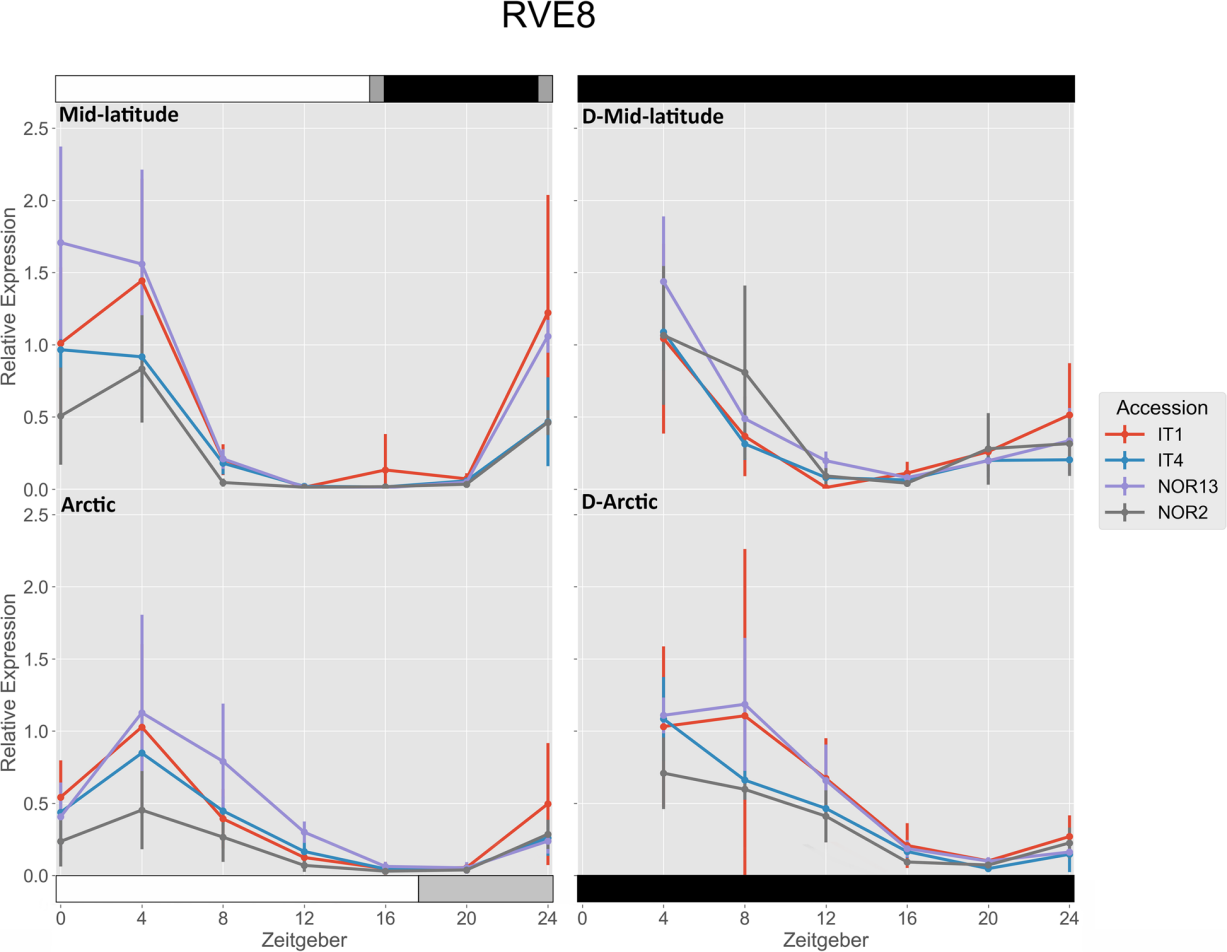
Relative expression of *FvRVE8*. The photoperiod conditions are indicated by the bars on the top and bottom, where the white bars indicate photosynthetic light, gray bars indicate non-photosynthetic light, and black bars indicate total darkness. ‘D-‘ represents when the plants were under total darkness for free-running rhythms. Each point for the light treatments represents the mean ± std of the 2^-ΔCq^ values of three biological replicates over two consecutive 24 hour periods, and each point for the free-running rhythms is over one 24 hour period

IT4 and NOR13 had significantly higher expression of *FvRVE8* under the mid-latitude photoperiod compared to the Arctic photoperiod at ZT 0 (Fig. 3). Under the mid-latitude photoperiod, NOR13 had significantly higher expression than IT4 and NOR2 at ZT 0. Under the Arctic photoperiod, IT1 and NOR13 had significantly higher expression than NOR2 at ZT 4. Only NOR2 had a significantly higher free-running rhythm after the mid-latitude photoperiod at ZT 8.

**Fig. 4:**
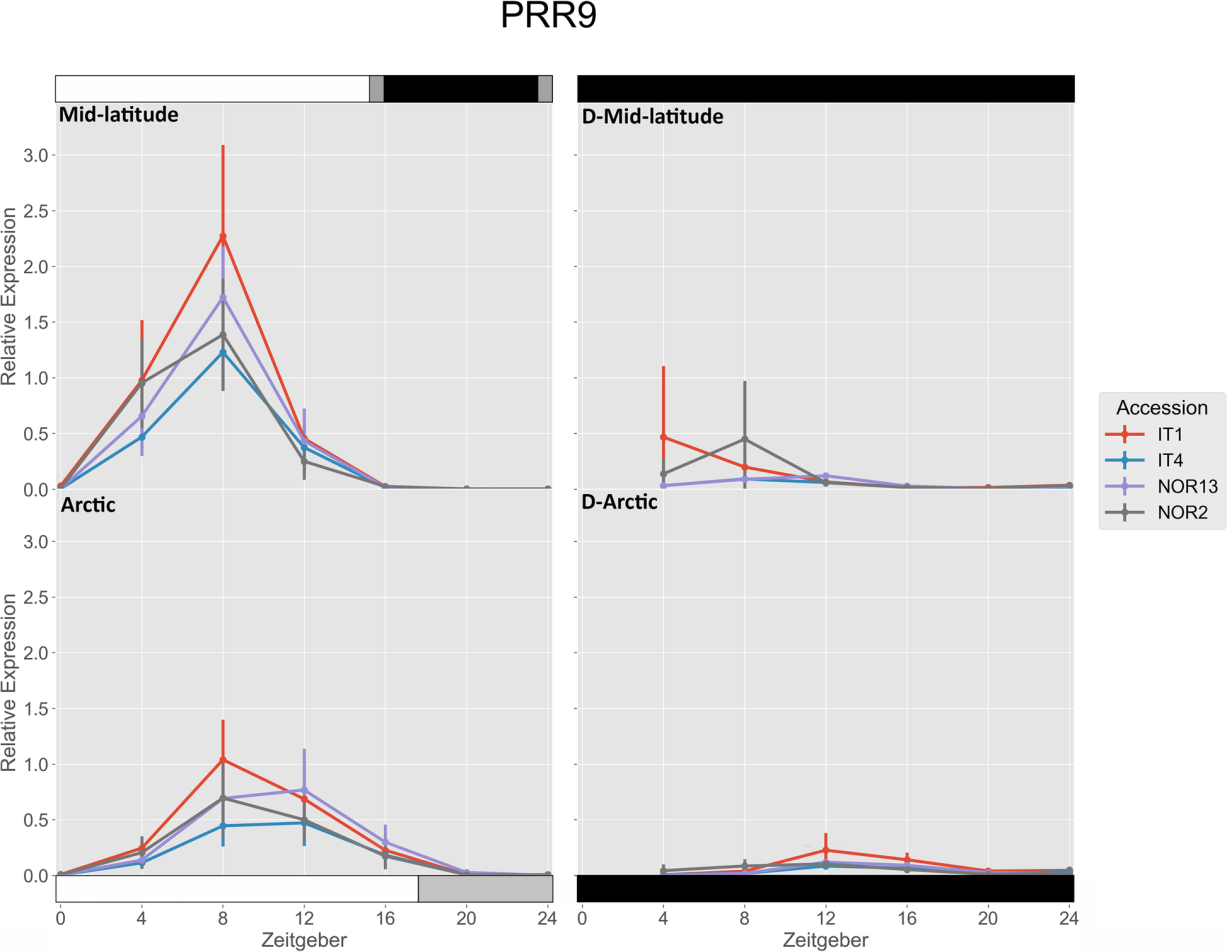
Relative expression of *FvPRR9*. The photoperiod conditions are indicated by the bars on the top and bottom, where the white bars indicate photosynthetic light, gray bars indicate non-photosynthetic light, and black bars indicate total darkness. ‘D-‘ represents when the plants were under total darkness for free-running rhythms. Each point for the light treatments represents the mean ± std of the 2^-ΔCq^ values of three biological replicates over two consecutive 24 hour periods, and each point for the free-running rhythms is over one 24 hour period

All accessions had significantly higher expression of *FvPRR9* under the mid-latitude photoperiod compared to the Arctic photoperiod at ZT 4 and ZT 8 (Fig. 4). Under the mid-latitude photoperiod, IT1 had significantly higher expression than IT4 and NOR2 at ZT 8. Under the Arctic photoperiod, IT1 had significantly higher expression than IT4 at ZT 8. Free-running rhythms after the mid-latitude photoperiod were significantly lower for IT4, NOR2, and NOR13 at ZT 4, and for all accessions at ZT 8. Free-running rhythms after the Arctic photoperiod were significantly lower for all accessions at ZT 8.

**Fig. 5:**
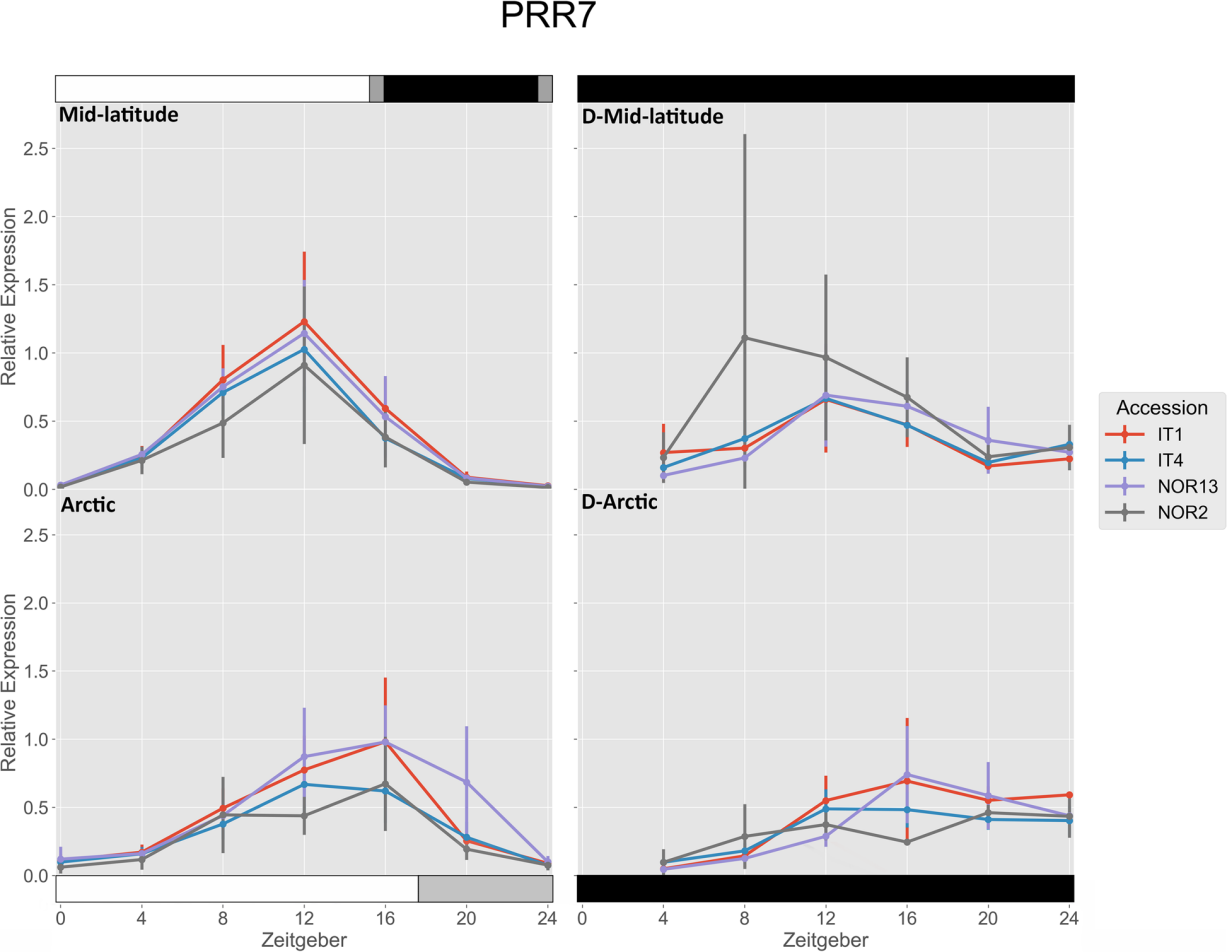
Relative expression of *FvPRR7*. The photoperiod conditions are indicated by the bars on the top and bottom, where the white bars indicate photosynthetic light, gray bars indicate non-photosynthetic light, and black bars indicate total darkness. ‘D-‘ represents when the plants were under total darkness for free-running rhythms. Each point for the light treatments represents the mean ± std of the 2^-ΔCq^ values of three biological replicates over two consecutive 24 hour periods, and each point for the free-running rhythms is over one 24 hour period

IT4 had significantly higher expression of *FvPRR7* under the mid-latitude photoperiod compared to the Arctic photoperiod at ZT 12, while NOR13 had significantly higher expression under the Arctic photoperiod at ZT 20 compared to the mid-latitude photoperiod (Fig. 5). There were no significant differences in free-running rhythms after either photoperiod.

**Fig. 6:**
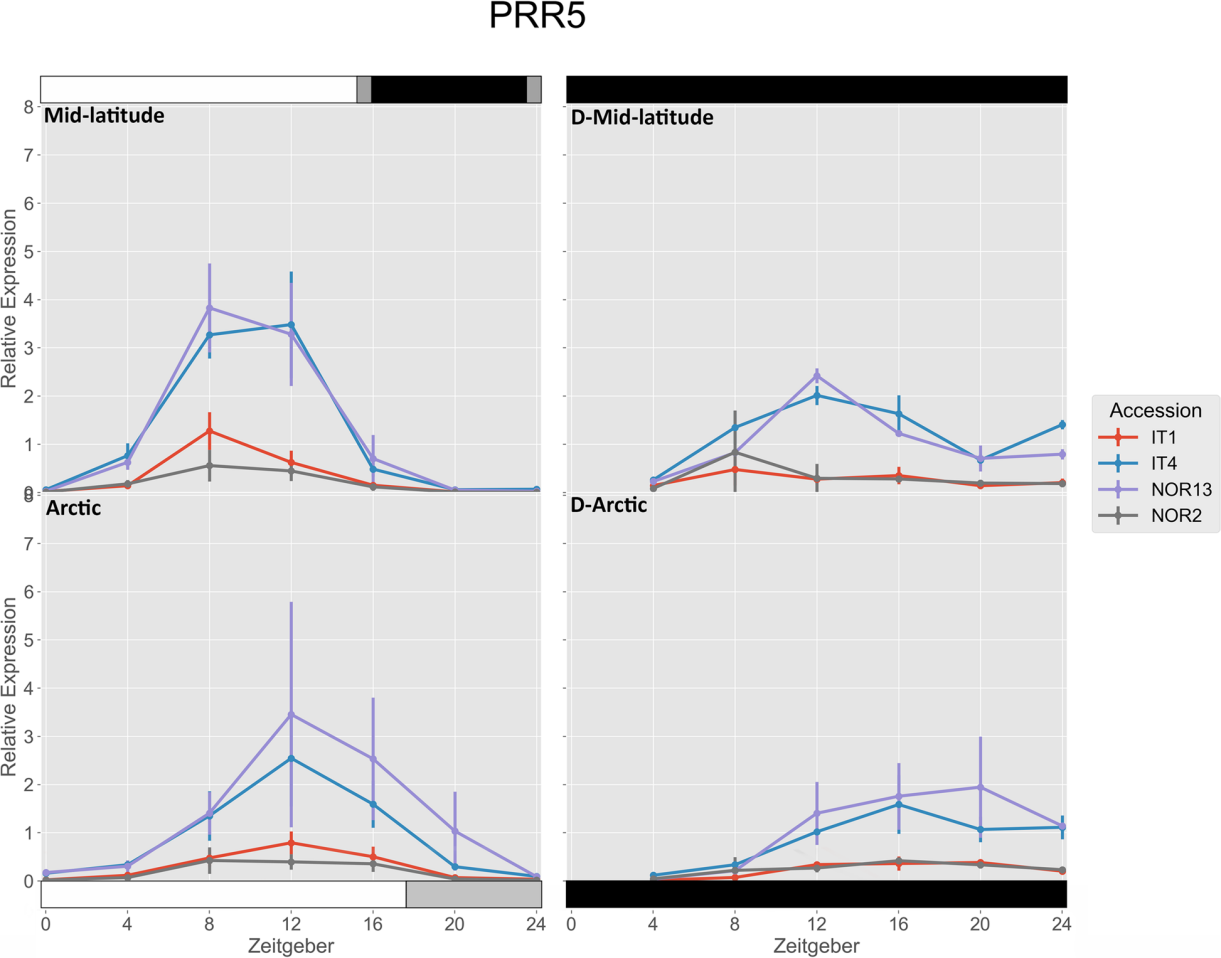
Relative expression of *FvPRR5*. The photoperiod conditions are indicated by the bars on the top and bottom, where the white bars indicate photosynthetic light, gray bars indicate non-photosynthetic light, and black bars indicate total darkness. ‘D-‘ represents when the plants were under total darkness for free-running rhythms. Each point for the light treatments represents the mean ± std of the 2^-ΔCq^ values of three biological replicates over two consecutive 24 hour periods, and each point for the free-running rhythms is over one 24 hour period

IT1, IT4, and NOR13 had significantly higher expression of *FvPRR5* under the mid-latitude photoperiod compared to the Arctic photoperiod at ZT 8, while IT4 was significantly higher under the Arctic photoperiod at ZT 16 compared to the mid-latitude photoperiod (Fig. 6). Under the mid-latitude photoperiod, NOR13 and IT4 had significantly higher expression than NOR2 and IT1 at ZT 8 and ZT 12. Under the Arctic photoperiod, NOR13 had significantly higher expression than NOR2 and IT1 at ZT 12 and ZT 16, and IT4 had significantly higher expression than NOR2 and IT1 at ZT 12. Free-running rhythms after the mid-latitude photoperiod were significantly lower for IT1, IT4, and NOR13 at ZT 8.

**Fig. 7:**
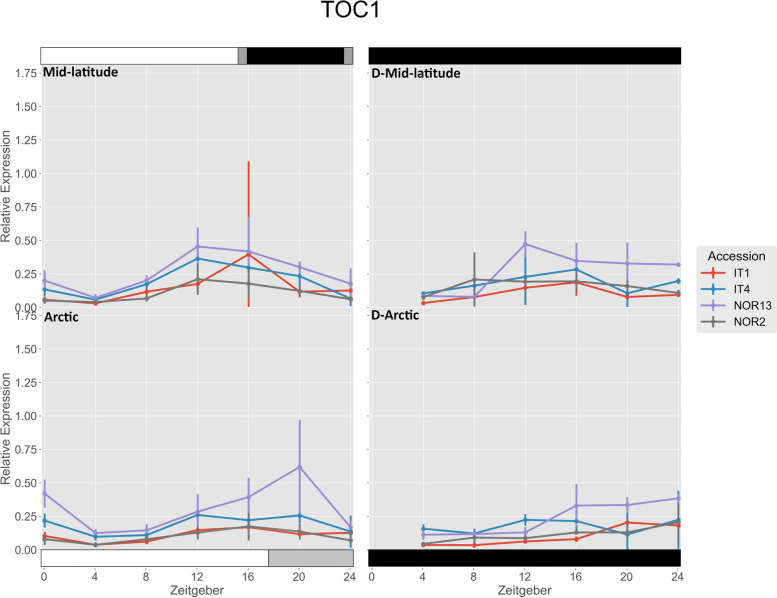
Relative expression of *FvTOC1* . The photoperiod conditions are indicated by the bars on the top and bottom, where the white bars indicate photosynthetic light, gray bars indicate non-photosynthetic light, and black bars indicate total darkness. ‘D-‘ represents when the plants were under total darkness for free-running rhythms. Each point for the light treatments represents the mean ± std of the 2^-ΔCq^ values of three biological replicates over two consecutive 24 hour periods, and each point for the free-running rhythms is over one 24 hour period

There were no significant differences in expression of *FvTOC1* within the accessions between the two treatments, or for any free-running rhythms (Fig. 7). Under the Arctic photoperiod, NOR13 had significantly higher expression than IT1 and NOR2 at ZT 0, and all other accessions at ZT 20.

**Fig. 8:**
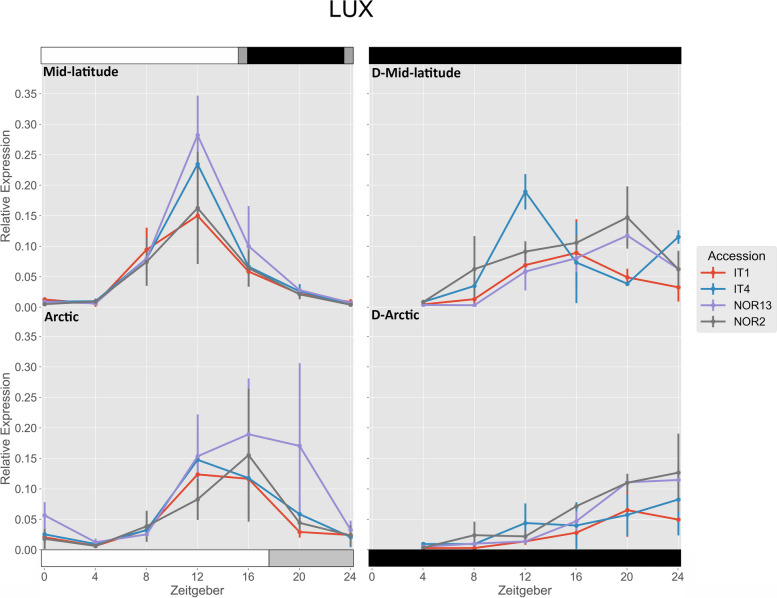
Relative expression of *FvLUX*. The photoperiod conditions are indicated by the bars on the top and bottom, where the white bars indicate photosynthetic light, gray bars indicate non-photosynthetic light, and black bars indicate total darkness. ‘D-‘ represents when the plants were under total darkness for free-running rhythms. Each point for the light treatments represents the mean ± std of the 2^-ΔCq^ values of three biological replicates over two consecutive 24 hour periods, and each point for the free-running rhythms is over one 24 hour period

IT4 and NOR13 had significantly higher expression of *FvLUX* under the mid-latitude photoperiod compared to the Arctic photoperiod at ZT 12, while NOR13 was significantly higher under the Arctic photoperiod at ZT 20 compared to the mid-latitude photoperiod (Fig. 8). Under the mid-latitude photoperiod, NOR13 had significantly higher expression than IT1 and NOR2 at ZT 12, and IT4 had significantly higher expression than IT1 at ZT 12. Under the Arctic photoperiod, NOR13 had significantly higher expression than all other accessions at ZT 20. Free-running rhythms after the mid-latitude photoperiod were significantly lower for IT1 at ZT 8 and ZT 12, and NOR13 at ZT 12, but significantly higher for NOR2 at ZT 20 and IT4 at ZT 24. Free-running rhythms after the Arctic photoperiod were significantly lower for IT1, IT4, and NOR13 at ZT 12, as well as for IT1 at ZT 16. The free-running rhythms were also significantly higher for IT4 at ZT 12 after the mid-latitude photoperiod compared to after the Arctic photoperiod.

### Differences in phytohormone accumulation under the Arctic photoperiod, mid-latitude photoperiod, and free-running rhythms

**Fig. 9:**
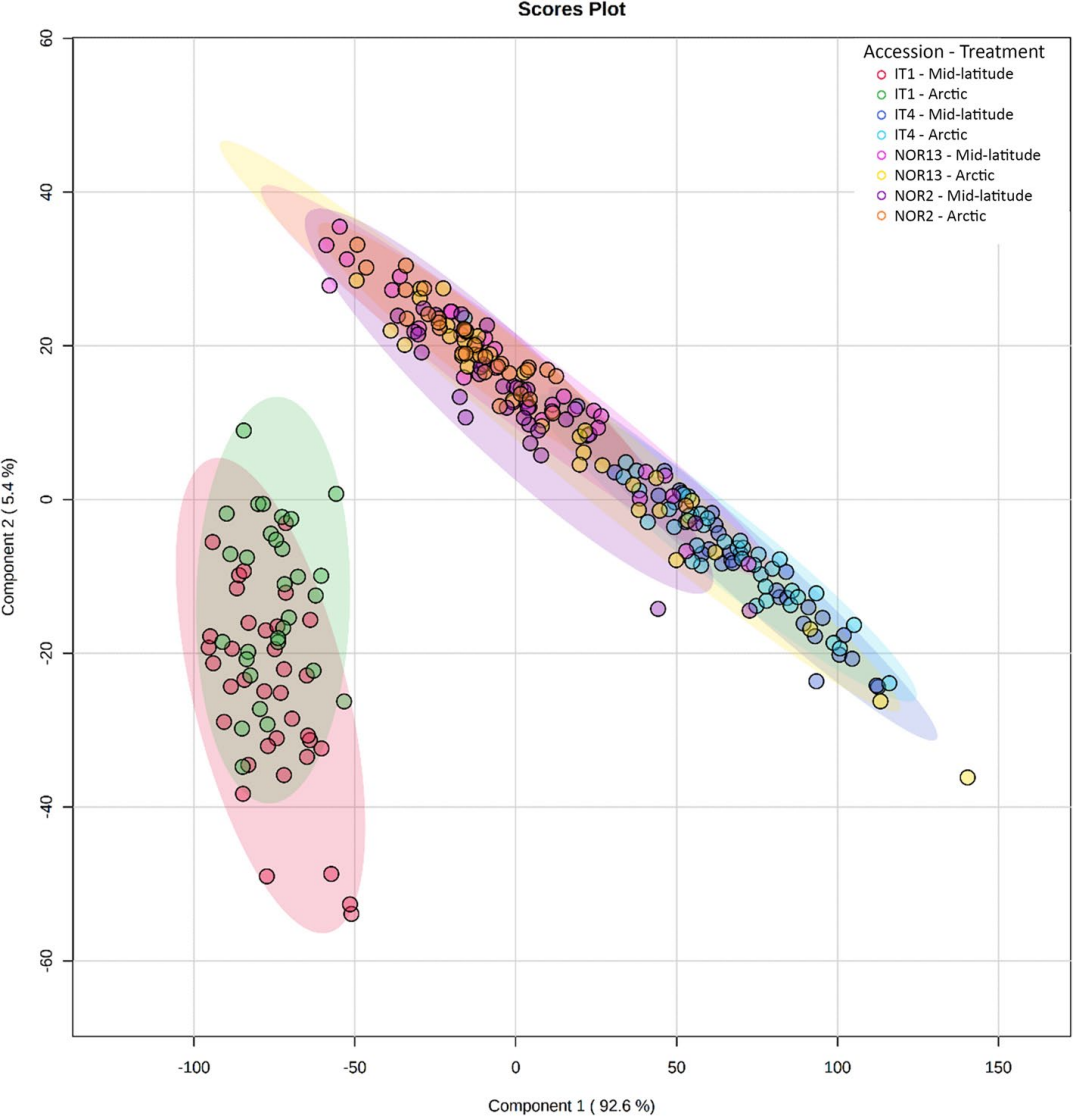
Difference in phytohormone accumulation between *Fragaria vesca* accessions. Partial Least Squares discriminant analysis (PLS-DA) scores plots of components one and two, comparing all phytohormones accumulating under the different daylength treatments in strawberry leaves. Colored ovals represent 95% confidence intervals. Colored dots represent individual samples: 92.6% and 5.4% are the scores of component 1 and component 2, respectively, in the PLS-DA analysis

A partial least squares discriminant analysis (PLS-DA) of all measured phytohormones together demonstrated marked separation of IT1 from the other accessions under both photoperiods (Fig. 9). Although there was no clear diurnal trend in any of the analyzed phytohormone levels (Suppl. Figure 1), significant differences in individual phytohormone level between the accessions were found.

**Fig. 10:**
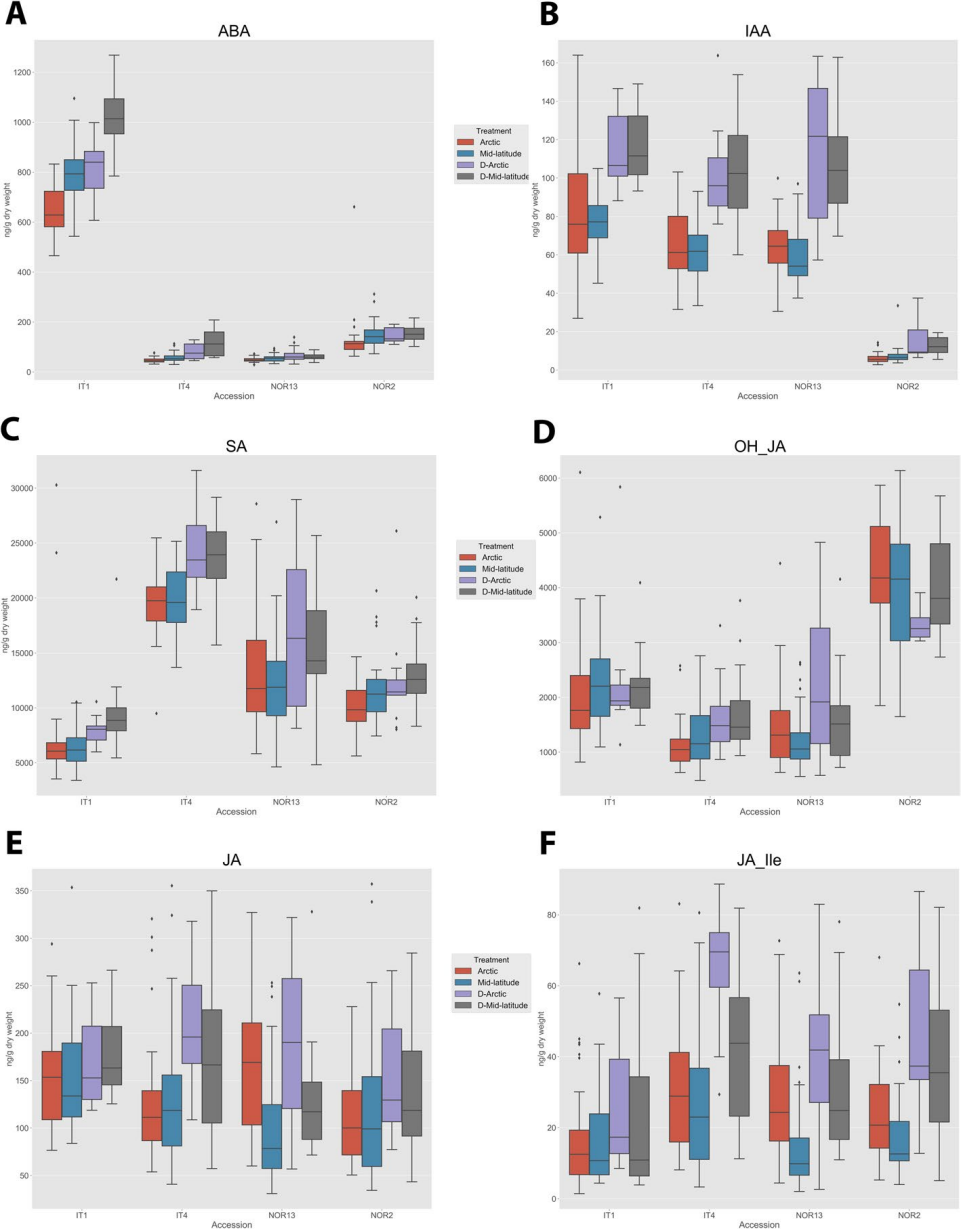
Phytohormone levels in the four *Fragaria vesca* accessions during the different light treatments. Abscisic acid (ABA) (**A**), auxin (IAA) (**B**), salicylic acid (SA) (**C**), hydroxyjasmonic acid (OH-JA), a jasmonate catabolite (**D**), jasmonic acid (JA) (**E**), jasmonic acid isoleucine-conjugate (JA-Ile) (**F**). Light conditions as indicated in Fig. 11. Shown are the means ± std of all samples taken over the 24 or 48 h period

Abscissic acid (ABA) levels were the highest in IT1 for all treatments (*p* = 0.001, Fig. 10A), while auxin (IAA) levels were the lowest in NOR2 (*p* = 0.001, Fig. 10B). Salicylic acid (SA) levels were the lowest in IT1 for all treatments (*p* = 0.001) while IT4 had the highest levels (*p* = 0.001, Fig. 10C). The jasmonate catabolite, hydroxyjasmonic acid (OH-JA), was highest in NOR2 (*p* = 0.001, Fig. 10D) and accumulated in much higher concentrations than JA (Fig. 10E). JA had no significant differences in accumulation at all. JA-Ile was only significantly higher when moved to constant darkness from the Arctic photoperiod (D-Arctic) in IT4 compared to IT1 (*p* = 0.001) and NOR13 (*p* = 0.04, Fig. 10F). All significant difference values for phytohormone levels are listed in Suppl. Table 4.

## Discussion

This study investigated whether seven core circadian clock genes and various phytohormones and derivatives of jasmonate in four different woodland strawberry accessions were affected by the differences in photoperiod corresponding with Arctic and mid-latitude daylength conditions that differ by 3h of photosynthetic light and a lack of darkness during the night in the Arctic (midnight sun).

### Circadian rhythms have lower amplitudes under Arctic photoperiods

Clock gene expression varied depending on both experienced photoperiod and endogenous factors within specific accessions. The Arctic photoperiod induced lower expression in IT4 and NOR13 for six of the seven genes (all but *FvTOC1*), in IT1 for three genes (*FvLHY, FvPRR9*, and *FvPRR5*), and in NOR2 for one gene (*FvPRR9*). IT1 had higher expression than all other accessions of *FvLHY* and *FvPRR9* under both photoperiods, while NOR13 had higher expression of *FvRVE8, FvTOC1*, and *FvLUX* under both photoperiods. The amplitudes of free-running rhythms for some genes were also influenced by both the previously exposed photoperiod and specific accession. IT1 and IT4 initially had higher free-running rhythms of *FvLHY* after the Arctic photoperiod compared to the mid-latitude photoperiod, but after the mid-latitude photoperiod, free-running expression for three of the accessions began to rise again after 24 h, whereas they didn’t after the Arctic photoperiod. IT4 had a higher free-running expression of *FvLUX* at ZT 12 after the mid-latitude photoperiod compared to the Arctic photoperiod, though free-running expression also appeared to lose the 24-hour rhythmicity for this accession. The attenuation of overt circadian rhythms has been observed in animals adapted to Arctic environments [27], but never in plants. Given that this phenomenon was observed in at least one gene in all accessions, including those native to middle latitudes, this suggests that reduced amplitudes of circadian rhythms under Arctic photoperiods could potentially offer some functional benefits to plants. However, it is important to note that this observation was limited to a two-week period, which could simply reflect the plasticity of the clock in response to short-term changes. Further research would be necessary to understand the long-term adjustment of the clock and its impact on physiological functions. The circadian genes here were chosen for their specific roles in the plant clock system. Both *FvRVE8* and *FvLHY* had a strong morning phase of expression, even in the Arctic daylength which had low levels of non-photosynthetic light at night, emphasizing the dependence on the predictable onset of photosynthetic light at dawn for timing of expression. While *LHY* is a key repressor of all other genes in the clock [13] *RVE8* is a direct transcriptional activator of evening-phased genes as well as involved in temperature compensation and light signaling [28]. The transcription profiles of these genes signifies their essential role in regulating the morning phase of the circadian clock.

*FvPRR9*, *FvPRR7*, and *FvPRR5* had roughly similar phases of expression at mid-day in *F. vesca*, which was also found in Chen et al. [12], though, here expression was lower under the Arctic photoperiod and the duration of expression was slightly longer. In *Arabidopsis*, in contrast, the *PRR*’s have been identified to express in sequential waves after dawn starting with *PRR9*, followed by *PRR7*, *PRR5*, *PRR3*, and ending with *TOC1* in the evening [29]. These mid-day-phased genes act as transcriptional repressors of *CCA1* and *LHY* in *Arabidopsis* [30], and coincided with the timing of decreased *LHY* expression in *F. vesca* here, confirming similarities in clock gene functions across higher plants [31].

*FvTOC1*’s phase of expression occurred throughout the evening and into the morning, but had the lowest expression at ZT 4 when *FvLHY* had the highest expression, providing supportive evidence of reciprocal repression between *TOC1* and morning genes [10]. *FvTOC1*’s extended phase of expression from afternoon into early morning has been reported in previous studies and aligns with *TOC1*’s close association with ABA to maintain cellular homeostasis during drought stress via processes such as stomatal closure, which largely occur at night [29, 32–34]. *LUX* along with the other components of the Evening Complex (EC), directly regulate *PRR9* in *Arabidopsis*, as well as play an important role in growth in the early evening, corresponding with the evening expression for *FvLUX* observed here [17, 35]. The EC regulates the circadian gating of hypocotyl growth in the early evening by repressing the expression of phytochrome interacting factors *PIF4* and *PIF5* that help regulate plant responses to differential red (R), far-red (FR), and blue light throughout the day, and are not activated again until the following morning by interacting with *LHY* [17, 36]. While here the evening light was only represented by 3 µmol m^–2^ s^−1^ of white light, not FR light, all clock components still behaved in ways indicating they perceived the difference between the photosynthetic light and non-photosynthetic light representative of twilight. Thus, the components in the clock system are directly dependent on the proper transcriptional timing of all other transcription factors in the TTFL to stay in sync with the external photoperiod, even when the light only fluctuates in its photosynthetic quality. This ensures proper timing of biological functions, hormone signaling, and metabolic processes in response to multiple abiotic and biotic factors [37]. However, it is yet to be determined whether the reduction in amplitude of circadian clock gene activity observed under Arctic photoperiods can be replicated in natural field conditions and warrants further studies.

### Phytohormone accumulation is dependent upon endogenous factors

Considering that the connection between the circadian clock and hormone signaling is crucial for plant growth and development, and to enhancing fitness in plants [38], the differences in experienced photoperiod or population origin were hypothesized to influence phytohormone accumulation. Phytohormone accumulation was primarily determined by genetic factors of the specific accessions rather than the experienced photoperiod or latitude of origin. Here, the accession from Tenno, Italy (IT1, 45°93’N) was identified as an accession that best discriminates between all accessions, very likely due to much higher levels of abscisic acid (ABA), but low levels of salicylic acid (SA) under all treatments, including complete darkness, while the other accession from Salorno, Italy (IT4, 46°23’N), had higher levels of SA, and the two Norwegian accession had moderate levels of SA. Since LHY interacts with the biosynthesis pathways of ABA [21], this may be the reason for IT1’s consistently higher expression of both *FvLHY* and ABA. Increased ABA/SA ratios were observed in drought-stressed *Brassica napus* (rapeseed) [39], however our plants were not exposed to any known stress factors (biotic or abiotic), thus this increase may be related to adaptations from the original environment. Additionally, there was no rhythmic accumulation of any of the phytohormones, which was unexpected as ABA [21], jasmonic acid (JA) [22], jasmonates, and salicylates [40] have been reported to accumulate rhythmically in *Arabidopsis*.

The accession from Alta, Norway (NOR2) had extremely low levels of indole-3-acetic acid (IAA) but high levels of the jasmonate catabolite, hydroxyjasmonic acid (OH-JA). IAA signal transduction is regulated by multiple steps of the circadian clock, where high auxin inducibility was correlated with high-amplitude circadian rhythms [23]. NOR2 had relatively low amplitudes of expression for several clock genes, which may explain NOR2’s low levels of IAA.

There was no significant difference in JA accumulation, though in a recent study, JA accumulation was significantly higher in bilberry (*Vaccinium myrtillus*) leaves under constant light (24 h) versus a 12 h (12 L/12 D) photoperiod [24]. It can be postulated that the lack of difference here may be due to the similarities in amount of photosynthetic light. Plants grown under the Arctic daylength received only 3 h longer photosynthetic light, and still experienced a 6 h period of non-photosynthetic light (twilight), which would be more typical of the early and late growing seasons, when herbivore-resistance induced by jasmonate levels would be less necessary than in peak summer. Additionally, the low levels of jasmonic acid-isoleucine conjugate (JA-Ile) observed here are typical of plants in the absence of stress [24]. Thus, the hypothesis that biosynthesis of defense-related phytohormones is promoted by high-latitude summer photoperiods may still hold true for *F. vesca* and would need to be investigated under 24 h photosynthetic light. There was only one observable difference in plant growth for NOR13, which was lower under the Arctic photoperiod, but this disparity did not cause any discernable effect on clock genes or phytohormone levels. However, we cannot exclude intrinsic determinations of phytohormones level in the different accessions, which is not unlikely as actually none of the light treatments caused significant changes within an accession. Therefore, the observed differences in endogenous phytohormone levels have a much more complex role in various physiological and biochemical processes than can be elucidated in this study.

### Relation between changes in photoperiod and physiological responses for future crop production

Climate change is causing a geographic shift for many species as land at northern latitudes becomes more habitable, however the experienced photoperiod may constrain physiological responses (e.g., early flowering) that are important for crop production [41]. It has been suggested that manipulation of the circadian clock may be a strategy to overcome light-related environmental changes due to its direct control of many developmental and metabolic processes [42]. However, most studies investigating the influence of photoperiod on the diurnal rhythm of gene expression in plants typically classify differences in daylength by long days (14–16 h of light) and short days (8–10 h of light) with corresponding dark periods for a 24 h cycle [43–45]. This is because the differences between short-days and long-days are key determining factors for circadian regulation of floral induction [31, 46]. Yet, this does not address how plants will respond to the differences in light conditions between middle and high latitudes. Since *F. vesca* has a natural geographic range that spans from 37°N to 70°N, and shares a significant amount of sequence identity with the economically important cultivated strawberry, this makes it useful when considering transcriptional studies that can be related to agricultural improvement [3–5]. Accession-specific differences have been observed for flowering responses in *F. vesca*, where a high-latitude population from Alta had an obligatory vernalization requirement but no vernalization was needed for flowering of other high-latitude populations [47, 48]. However, photoperiodic responses like flowering, are complex traits with many loci that have multiple regulatory factors in response to changes in daylength [49, 50]. Therefore, while it has been shown here that *F. vesca* accessions from Italy and Norway can acclimate their circadian clocks to both Arctic and mid-latitudinal photoperiods, the effects of the lower amplitudes of expression on physiological functioning requires further investigation. Understanding how species adapt circadian regulation to different photoperiods can reveal important selection pressures on cellular and physiological responses that may be of crucial importance for future crop production [25, 51, 52].

## Conclusions

Our study shows that the circadian clock in *Fragaria vesca* accessions from Italy and Northern Norway can acclimate to both Arctic and mid-latitude photoperiods. Under the Arctic photoperiod, certain clock genes exhibited reduced amplitudes compared to those under the mid-latitude photoperiod. However, the extent of these variations depended on specific accessions’ endogenous factors, indicating that the amplitude of circadian rhythms is influenced by both environmental and genetic factors. In contrast, the genetic factors of specific accessions primarily determined the accumulation of phytohormones, rather than the experienced photoperiod. These differences suggest the existence of conserved adaptations from their original environments.

## Materials and methods

### Plant materials and treatments

Four *Fragaria vesca* accessions were obtained from the University of Helsinki (Dr. Timo Hytönen’s lab). Two originated from Italy; IT1 (Tenno, Ville del Monte, Lago di Tenno): 45°93’N, 10°81’E; and IT4 (Da Salorno, Pochi, Alto, Adige, Italy): 46°23’N, 11°23’ E, and two from Northern Norway; NOR2 (Alta, Leirbukta): 69°10’ N, 23°67’ E, and NOR13 (Indre Nordnes): 69°53’ N, 20°38’ E. Clones were propagated from each of the four accessions in 1:1 [v/v] peat soil and perlite under a 12 h photoperiod (18 °C) prior to light experiments. Clones were grown for 18 days at a constant temperature of 18 °C in separate growth chambers under two daylength treatments: 18 h photosynthetic light/6 h twilight for the Arctic photoperiod, or 15 h photosynthetic light/1 h twilight/8 h dark for the mid-latitude photoperiod (Fig. 7).

**Fig. 11:**
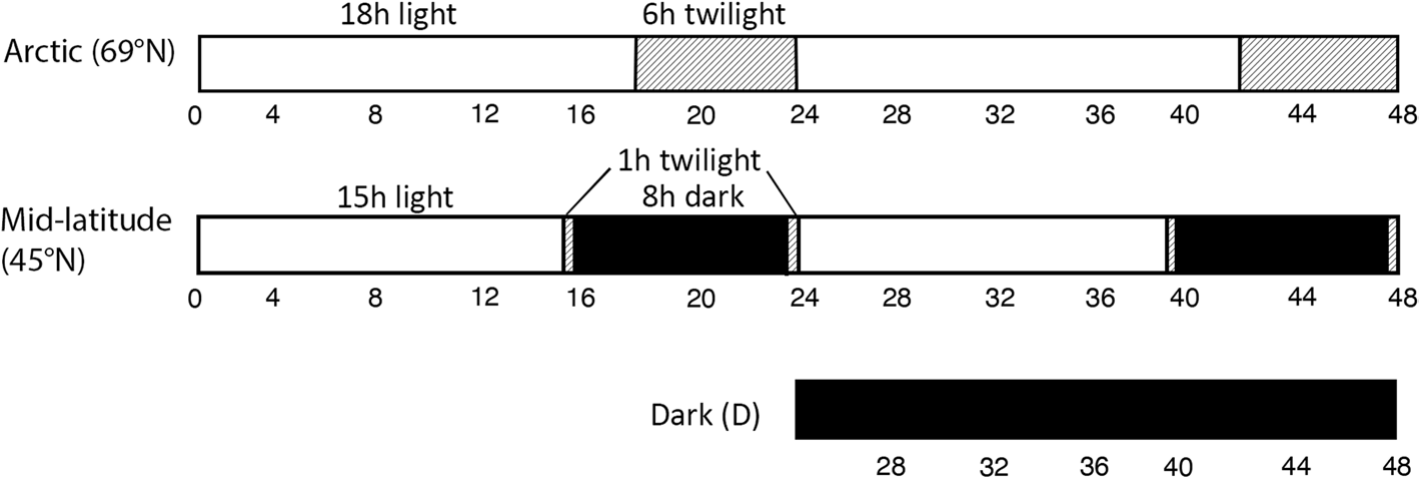
Photoperiod treatments. The two photoperiod treatments for the Arctic photoperiod (18 h photosynthetic light, 6 h twilight), and mid-latitude photoperiod (15 h photosynthetic light, 1 h total twilight, 8 h dark).The times indicate the 13 timepoints (zeitgebers, ZT) of sampling

Light conditions were applied using fluorescent tubes (PHILIPS Cool White MASTER TL-D Super 80 58 W/840, Eindhoven, The Netherlands) to simulate photosynthetic light and light emitting diodes (LED, PHILIPS Softone 18 W) to simulate non-photosynthetic light/twilight. Total photosynthetic photon flux density (PPFD) was adjusted in each treatment to give roughly the same amount of total photosynthetic light over the two different daylengths and measured using a quantum sensor (LI-1000, LI-COR Inc., USA). The Arctic photoperiod had a PPFD of 200 µmol m^–2^ s^–^ for the photosynthetic light, the mid-latitude photoperiod had a PPFD of 243 µmol m^–2^ s^–^, and the non-photosynthetic light for both treatments had a PPFD of 3 µmol m^–2^ s^–^. Leaves from three biological replicates of each accession were collected at 4 h intervals from Zeitgeber time 0 (ZT 0) to ZT 48 (*N* = 114 per accession), put directly into liquid N_2_ and stored at -80 °C until RNA extraction. At ZT 24, half of the remaining plants from each treatment (*N* = 36 per accession) were transferred to total darkness and concurrently sampled to observe free-running rhythms.

### RNA extraction and cDNA synthesis

Total RNA was extracted from finely ground leaf tissue following a modified protocol from Jaakola, Pirttilä [53] using the E.Z.N.A Total RNA Kit I (Omega Bio-tek Inc., USA, 2020). DNA was removed by RNase-free DNase (Qiagen, US) according to manufacturer’s instructions. The purity and concentration of RNA samples were assessed using Nanodrop 2000 spectrophotometer (ThermoFisher, USA). RNA integrity was assessed using 1.2% (w/v) agarose gel electrophoresis. cDNA was synthesized from 0.75 µg total RNA in a final reaction volume of 10 µL using Superscript IV First-Strand cDNA Synthesis Reaction protocol (Invitrogen, Carlsbad, CA, USA) according to manual instructions and stored at -20 °C until use.

### Primer design

Seven circadian genes, one universally conserved protein marker, and two reference genes were selected based on previous studies in *F. vesca.* Five gene primer sequences (*FvLHY, FvPRR9, FvPRR7, FvPRR5, and FvRVE8*) and one reference gene primer sequence (*FvGAPDH)* were selected from Chen et al. [12]. Gene ID’s for *FvTOC1, FvLUX*, and *FvMSI1* were searched in the *F. vesca* genome database (*F. vesca* Whole Genome v1.0 (build 8) Assembly & Annotation) at the Rosaceae genome database (GDR, https:/www.rosaceae.org) to get the genomic sequences. *FvMSI1* weas subject to primer design testing in a separate unpublished scientific report. NCBI Primer-BLAST (https://www.ncbi.nlm.nih.gov/tools/primer-blast/) was used to design specific primers for remaining genes according to the standard primer parameters. Primer sequences are listed in Suppl. Table 1.

### Quantitative reverse transcription PCR (qRT-PCR)

qRT-PCR assays were performed using a CFX96 Touch Real-Time PCR System (Bio-Rad, Hercules, CA, USA) to analyze RNA expression. Each 15 µL reaction mixture contained 1 µL of 10^−1^ diluted cDNA template, 7.5 µL of SsoFast™ EvaGreen Supermix (Bio-Rad), 3.5 µL of sterile H_2_O, and 1.5 µL (5 µM) of each forward and reverse primer. Amplification cycling conditions were as follows: 1 denaturation cycle of 95 °C for 2 m, followed by 40 cycles of 95 °C for 5 s, 60 °C for 30 s, ending with 1 cycle for the melt curve analysis from 65 to 95 °C in 0.5 °C increments for 5 s to verify analytical specificity. The sample-maximization method [54] was used to minimize technical (run-to-run) variation. A no-template control (NTC) was included for each gene assay to account for contamination or non-specific gene products.

### Expression validation

Primer pair specificity was confirmed via single peak detection with no signals on the negative controls in the melt curve analysis. All tested genes had efficiency (E%) values ranging from 89 to 117%, with regression coefficient (R^2^) varying from 0.96 to 1.00.

### Phytohormone measurements

Remaining ground leaf tissue was lyophilized for 4 days. Between 10 and 30 mg of dry leaf material was extracted in 1.5 ml methanol containing 60 ng D4-SA (Santa Cruz Biotechnology, USA), 60 ng D6-JA (HPC Standards GmbH, Germany), 60 ng D6-ABA (Santa Cruz Biotechnology, USA), 12 ng D6-JA-Ile (HPC Standards GmbH), and D5-indolacetic acid (D5-IAA, OlChemIm s.r.o., Olomouc, Czech Republic) as internal standards. Samples were agitated on a horizontal shaker at room temperature for 10 min. The homogenate was mixed for 30 min and centrifuged at 13,000 rpm for 20 min at 4 °C and the supernatant was collected. The homogenate was re-extracted with 500 µl methanol, mixed and centrifuged and the supernatants were pooled. The combined extracts were evaporated under reduced pressure at 30 °C and dissolved in 500 µl methanol.

Phytohormone analysis was performed by LC-MS/MS as in [55, 56] on an Agilent 1260 series HPLC system (Agilent Technologies) coupled to a tandem mass spectrometer QTRAP 6500 (SCIEX, Darmstadt, Germany). Details of the instrument parameters and response factors for quantification can be found in Suppl. Table 5.

Indolacetic acid was quantified using the same LC-MS/MS system with the same chromatographic conditions but using positive mode ionization with an ion spray voltage at 5500 eV. Multiple reaction monitoring (MRM) was used to monitor analyte parent ion → product ion fragmentations as follows: m/z 176 →130 (collision energy (CE ) 19 V; declustering potential (DP) 31 V) for indolacetic acid (IAA); m/z 181 →133 + m/z 181 →134 + m/z 181 →135 (CE 19 V; DP 31 V) for D5-indolacetic acid.

### Statistical analysis

The real-time qRT-PCR expression data and phytohormone data were analyzed using *Jupyter Notebook*. The 2^− ΔCq^ values were normalized to the geometric mean of the two reference genes following the ΔCq method [54] and grouped by ZT 0 - ZT 24. For each gene, the mean ± std of three biological replicates over two consecutive 24 h periods were plotted for each light treatment, and the mean ± std of three biological replicates over one 24 h period were plotted for each dark treatment. A three-way analysis of variance (ANOVA) and Tukey post-hoc test was performed for each individual gene using the statsmodels package to determine at which ZT there was a significant difference between the treatments within each accession (Suppl. Table 2), and at which ZT there was a significant difference between the accessions within each treatment (Suppl. Table 3). A two-way ANOVA and Tukey post-hoc test was performed for the phytohormone results to determine which clones had significant differences in accumulation between the treatments. Both the real-time qRT-PCR results and phytohormone results were confirmed by three biological repetitions. The scripts for the qPCR data analysis and phytohormone analysis are available from the corresponding author upon request.

### Supplementary Information

**Additional file 1: Supplementary Table 1.** List of genes, primers, source, and efficiency values (E%) used in this study. **Supplementary Table 2.** Significant differences between treatments within each accession. **Supplementary Table 3.** Significant differences within accessions within each treatment. **Supplementary Table 4.** Significant differences in phytohormone data between accessions within treatments. **Supplementary Table 5.** Details of analysis of phytohormones by LC-MS/MS [HPLC 1260 (Agilent Technologies)-QTRAP6500 (SCIEX)] in negative ionisation mode. **Supplementary Figure 1.** Average phytohormone levels within each treatment over a 24 h period.

## Data Availability

The datasets generated and analyzed during the current study are available in the Python-qPCR-analysis repository, https://zenodo.org/badge/latestdoi/359723030.
