# Supplementary material for: Acclimation of circadian rhythms in woodland strawberries (Fragaria vesca L.) to Arctic and mid-latitude photoperiods
Source: BMC Plant Biol. 2023 Oct 10;23:483. doi: 10.1186/s12870-023-04491-6 (PMC10563271; doi:10.1186/s12870-023-04491-6)

Supplementary Table 1. List of genes, primers, source, and efficiency values (E%) used in this study.

Gene	Gene ID	Full Name	Forward Primer (5'-3')	Reverse Primer (3'-5')	Source	E%
Target Genes						
FvLHY	gene18601	Late Elongated Hypocotyl	CACTCAGAAGCTGTGCAAGC	GTAACTGGACTCAGAAGCTG	Chen et al. 2018	91.36
FvPRR9	gene18611	Two-component response regulator-like PRR9	GTAGCAGGTGAGATGGCCAC	CAATGTGAGCTCTATGCACG	Chen et al. 2018	109.29
FvPRR7	gene18151	Two-component response regulator-like PRR 7	CTGCAGCAGCTCCACATTGC	CGAGGTCGCTGTTCTGCTAG	Chen et al. 2018	106.36
FvRVE8	gene18104	Reveille 8	GTCACAAGCTCATGTGCGCCA	CTGCTGAGCTATAGTCCGAAG	Chen et al. 2018	94.06
FvPRR5	gene12454	Two-component response regulator-like PRR 5	CTGCAATGGCAATGCAAGTC	GAAGGATCAGTAACTTGACG	Chen et al. 2018	112.02
FvTOC1/ PRR1	gene26055	Timing of CAB 1/ Two-component response regulator-like PRR1	CCGTCCGATCAGTCCTCTTC	CTCGACTCGTCGTTATCGCA	NCBI Primer-Blast	109.56
FvLUX/ PCL1	gene30204	Transcription factor Lux arrhythmo/ Phytoclock 1	ACTTGCAGAAGTACCGCCTC	CGCAACATGTGGTTGGTACG	NCBI Primer-Blast	114.43
Reference Genes						
FvGAPDH	gene07104	Glyceraldehyde-3-Phosphate Dehydrogenase	CAGAAGACTGTTGATGGACC	GCAGCCTTAATCTGGTCATAG	Chen et al. 2018	89.35
FvMSI1	gene03001	WD-40 repeat protein Multicopy Suppressor of IRA1	TCCCCACACCTTTGATTGCCA	ACACCATCAGTCTCCTGCCAAG	Mouhu et al. 2013	117.20

Supplementary Table 2. Significant differences between treatments within each accession.

LHY							
**Treatment_1**	**Treatment_2**	**Accession**	**ZT**	**meandiff**	**lower**	**upper**	**p-adj**
Mid-latitude	Arctic	IT1	0	7.23	1.46	13.01	0.002
		IT4	0	0.48	0.25	0.71	0.001
		NOR13	0	1.31	0.59	2.04	0.001
		IT1	4	6.95	1.50	12.39	0.002
		IT1	24	8.84	1.82	15.87	0.002
		IT4	24	0.46	0.14	0.77	0.001
		NOR13	24	0.85	0.01	1.69	0.043
Arctic	D-Arctic	IT1	4	7.82	1.53	14.10	0.002
		IT4	4	0.38	0.10	0.66	0.001
		NOR2	4	8.04	1.35	14.73	0.004
		IT1	8	7.99	1.91	14.08	0.001
		IT4	8	0.30	0.02	0.58	0.026
Mid-latitude	D-Mid-latitude	IT4	4	-0.31	-0.59	-0.03	0.017
		NOR2	8	-11.13	-17.82	-4.44	0.001
		IT1	24	10.06	2.21	17.92	0.001
		IT4	24	0.63	0.28	0.98	0.001
		NOR13	24	1.02	0.09	1.96	0.018
D-Arctic	D-Mid-latitude	IT1	8	-8.58	-15.60	-1.55	0.003
		IT4	8	-0.36	-0.68	-0.05	0.007

RVE8							
**Treatment_1**	**Treatment_2**	**Accession**	**ZT**	**meandiff**	**lower**	**upper**	**p-adj**
Mid-latitude	Arctic	IT4	0	0.53	0.22	0.84	0.001
		NOR13	0	1.30	0.47	2.13	0.001
Mid-latitude	D-Mid-latitude	NOR2	8	-0.76	-1.33	-0.20	0.001

PRR9							
**Treatment_1**	**Treatment_2**	**Accession**	**ZT**	**meandiff**	**lower**	**upper**	**p-adj**
Mid-latitude	Arctic	IT1	4	0.73	0.01	1.45	0.040
		IT4	4	0.35	0.07	0.64	0.002
		NOR2	4	0.75	0.28	1.22	0.001
		NOR13	4	0.52	0.07	0.96	0.007
		IT1	8	1.23	0.58	1.89	0.001
		IT4	8	0.78	0.50	1.07	0.001

Treatment_1	Treatment_2	Accession	ZT	meandiff	lower	upper	p-adj
		NOR2	8	0.69	0.22	1.16	0.001
		NOR13	8	1.03	0.59	1.47	0.001
Arctic	D-Arctic	IT1	8	-1.00	-1.80	-0.20	0.002
		IT4	8	-0.43	-0.75	-0.10	0.001
		NOR2	8	-0.61	-1.18	-0.04	0.024
		NOR13	8	-0.67	-1.16	-0.19	0.001
		IT4	12	-0.39	-0.76	-0.02	0.031
		NOR13	12	-0.65	-1.13	-0.16	0.001
Mid-latitude	D-Mid-latitude	IT4	4	0.44	0.11	0.76	0.001
		NOR2	4	0.82	0.24	1.39	0.001
		NOR13	4	0.62	0.10	1.15	0.005
		IT1	8	2.08	1.27	2.88	0.001
		IT4	8	1.14	0.81	1.46	0.001
		NOR2	8	0.94	0.37	1.51	0.001
		NOR13	8	1.63	1.11	2.16	0.001

PRR7

Treatment_1	Treatment_2	Accession	ZT	meandiff	lower	upper	p-adj
Mid-latitude	Arctic	IT4	12	0.36	0.01	0.70	0.032
		NOR13	20	-0.60	-1.14	-0.07	0.010

PRR5

Treatment_1	Treatment_2	Accession	ZT	meandiff	lower	upper	p-adj
Mid-latitude	Arctic	IT1	8	0.80	0.43	1.17	0.001
		IT4	8	1.92	0.82	3.02	0.001
		NOR13	8	2.42	0.38	4.46	0.005
		IT4	16	-1.11	-2.10	-0.12	0.013
Arctic	D-Arctic	IT4	12	-1.53	-2.90	-0.15	0.014
Mid-latitude	D-Mid-latitude	IT1	8	0.80	0.35	1.25	0.001
		IT4	8	1.92	0.55	3.29	0.001
		NOR13	8	3.00	0.59	5.41	0.002
		IT4	12	1.47	0.27	2.67	0.003

LUX

Treatment_1	Treatment_2	Accession	ZT	meandiff	lower	upper	p-adj
Arctic	Mid-latitude	IT4	12	0.09	0.02	0.15	0.001
		NOR13	12	0.13	0.02	0.24	0.010
		NOR13	20	-0.14	-0.26	-0.02	0.004
Arctic	D-Arctic	IT1	12	-0.11	-0.19	-0.03	0.001
		IT4	12	-0.10	-0.19	-0.02	0.003

		NOR13	12	-0.14	-0.28	0.00	0.047
		IT1	16	-0.09	-0.17	-0.01	0.011
Mid-latitude	D-Mid-latitude	IT1	8	0.08	0.00	0.16	0.047
		IT1	12	0.08	0.00	0.16	0.048
		NOR13	12	0.22	0.09	0.36	0.001
		NOR2	20	-0.13	-0.23	-0.02	0.006
		IT4	24	-0.11	-0.20	-0.02	0.005
D-Arctic	D-Mid-latitude	IT4	12	0.15	0.05	0.24	0.001

Supplementary Table 3. Significant differences within accessions within each treatment.

**LHY**							
**Treatment**	**Accession_1**	**Accession_2**	**ZT**	**meandiff**	**lower**	**upper**	**p-adj**
Arctic	IT1	IT4	0	-4.99	-6.97	-3.02	0.001
	IT1	NOR13	0	-4.75	-6.94	-2.56	0.001
	IT1	NOR2	0	-3.15	-5.22	-1.09	0.001
	IT1	IT4	4	-7.30	-9.37	-5.24	0.001
	IT1	NOR13	4	-6.85	-8.83	-4.88	0.001
	IT1	NOR2	4	-4.02	-6.00	-2.05	0.001
	NOR2	IT4	4	3.28	1.30	5.26	0.001
	NOR2	NOR13	4	2.83	0.94	4.71	0.001
	IT1	IT4	8	-3.02	-5.00	-1.05	0.001
	IT1	NOR13	8	-2.84	-4.73	-0.96	0.001
	NOR2	IT4	8	2.16	0.19	4.14	0.016
	NOR2	NOR13	8	1.98	0.10	3.86	0.027
	IT1	IT4	24	-3.09	-5.75	-0.42	0.007
	IT1	NOR13	24	-3.03	-5.69	-0.37	0.009
Mid-latitude	IT1	IT4	0	-11.75	-15.50	-7.99	0.001
	IT1	NOR13	0	-10.67	-14.63	-6.71	0.001
	IT1	NOR2	0	-7.73	-11.34	-4.11	0.001
	NOR2	IT4	0	4.02	0.63	7.41	0.005
	IT1	IT4	24	-11.47	-16.05	-6.90	0.001
	IT1	NOR13	24	-11.03	-15.60	-6.45	0.001
	NOR2	IT4	24	7.19	2.62	11.77	0.001
	NOR2	NOR13	24	6.75	2.18	11.32	0.001
	IT1	IT4	4	-14.36	-17.90	-10.81	0.001
	IT1	NOR13	4	-14.21	-17.96	-10.45	0.001
	IT1	NOR2	4	-10.75	-14.14	-7.36	0.001
	NOR2	IT4	4	3.60	0.21	7.00	0.023

RVE8							
**Treatment**	**Accession_1**	**Accession_2**	**ZT**	**meandiff**	**lower**	**upper**	**p-adj**
Arctic	IT1	NOR2	4	-0.58	-1.12	-0.03	0.028
	NOR13	NOR2	4	-0.67	-1.20	-0.15	0.001
	NOR13	NOR2	8	-0.52	-1.05	0.00	0.048
Mid-latitude	NOR13	IT4	0	0.74	0.00	1.48	0.047
	NOR13	NOR2	0	-1.20	-1.91	-0.49	0.001
	NOR13	NOR2	4	-0.73	-1.44	-0.02	0.039
**PRR9**							
**Treatment**	**Accession_1**	**Accession_2**	**ZT**	**meandiff**	**lower**	**upper**	**p-adj**
Arctic	IT1	IT4	8	-0.59	-1.00	-0.19	0.001
Mid-latitude	IT1	IT4	8	-1.04	-1.70	-0.39	0.001
	IT1	NOR2	8	-0.89	-1.51	-0.26	0.001
**PRR7**							
**Treatment**	**Accession_1**	**Accession_2**	**ZT**	**meandiff**	**lower**	**upper**	**p-adj**
Arctic	NOR13	NOR2	20	-0.49	-0.95	-0.03	0.021
**PRR5**							
**Treatment**	**Accession_1**	**Accession_2**	**ZT**	**meandiff**	**lower**	**upper**	**p-adj**
Arctic	IT4	IT1	12	1.75	0.28	3.23	0.004
	IT4	NOR2	12	-2.15	-3.78	-0.52	0.001
	NOR13	IT1	12	2.66	1.26	4.07	0.001
	NOR13	NOR2	12	-3.06	-4.63	-1.49	0.001
	NOR13	IT1	16	2.03	0.56	3.50	0.001
	NOR13	NOR2	16	-2.18	-3.81	-0.55	0.001
Mid-latitude	IT4	IT1	8	1.99	1.07	2.91	0.001
	IT4	NOR2	8	-2.71	-3.63	-1.79	0.001
	NOR13	IT1	8	2.55	1.57	3.53	0.001
	NOR13	NOR2	8	-3.27	-4.25	-2.29	0.001
	IT4	IT1	12	2.86	1.90	3.82	0.001
	IT4	NOR2	12	-3.04	-4.00	-2.07	0.001
	NOR13	IT1	12	2.66	1.74	3.58	0.001
	NOR13	NOR2	12	-2.83	-3.75	-1.91	0.001
**TOC1**							
**Treatment**	**Accession_1**	**Accession_2**	**ZT**	**meandiff**	**lower**	**upper**	**p-adj**
Arctic	NOR13	IT1	0	0.32	0.06	0.57	0.002
	NOR13	NOR2	0	-0.34	-0.60	-0.08	0.001
	NOR13	IT1	20	0.50	0.27	0.73	0.001
	NOR13	IT4	20	0.36	0.14	0.58	0.001
	NOR13	NOR2	20	-0.48	-0.70	-0.26	0.001
**LUX**							
**Treatment**	**Accession_1**	**Accession_2**	**ZT**	**meandiff**	**lower**	**upper**	**p-adj**

Arctic	NOR13	IT1	20	0.14	0.03	0.25	0.001
	NOR13	IT4	20	0.11	0.01	0.22	0.026
	NOR13	NOR2	20	-0.13	-0.23	-0.02	0.004
Mid-latitude	IT4	IT1	12	0.08	0.00	0.16	0.025
	NOR13	IT1	12	0.13	0.06	0.21	0.001
	NOR13	NOR2	12	-0.12	-0.20	-0.04	0.001

Supplementary Table 4. Significant differences in phytohormone data between accessions within treatments.

ABA						
**Treatment**	**Accession_1**	**Accession_2**	**meandiff**	**lower**	**upper**	**p-adj**
Arctic	IT1	IT4	-602.97	-655.14	-550.81	<0.001
	IT1	NOR13	-600.63	-652.80	-548.46	<0.001
	IT1	NOR2	-521.86	-575.11	-468.61	<0.001
	IT4	NOR2	81.12	27.87	134.36	<0.001
	NOR13	NOR2	78.77	25.52	132.02	<0.001
Mid-latitude	IT1	IT4	-740.37	-792.54	-688.20	<0.001
	IT1	NOR13	-744.24	-796.40	-692.07	<0.001
	IT1	NOR2	-650.68	-706.11	-595.25	<0.001
	IT4	NOR2	89.69	34.26	145.12	<0.001
	NOR13	NOR2	93.55	38.12	148.99	<0.001
D-Arctic	IT1	IT4	-733.11	-809.90	-656.32	<0.001
	IT1	NOR13	-747.22	-824.02	-670.43	<0.001
	IT1	NOR2	-669.93	-753.78	-586.08	<0.001
D-Mid-latitude	IT1	IT4	-905.31	-982.10	-828.52	<0.001
	IT1	NOR13	-960.13	-1036.92	-883.34	<0.001
	IT1	NOR2	-869.47	-946.26	-792.68	<0.001
	NOR13	NOR2	90.66	13.86	167.45	0.006
**IAA**						
**Treatment**	**Accession_1**	**Accession_2**	**meandiff**	**lower**	**upper**	**p-adj**
Arctic	IT1	IT4	-18.33	-32.98	-3.68	0.002
	IT1	NOR13	-19.25	-33.91	-4.60	0.001
	IT1	NOR2	-77.63	-92.59	-62.68	<0.001
	IT4	NOR2	-59.30	-74.26	-44.35	<0.001
	NOR13	NOR2	-58.38	-73.33	-43.42	<0.001
Mid-latitude	IT1	IT4	-15.66	-30.32	-1.01	0.023
	IT1	NOR13	-18.80	-33.46	-4.15	0.001

	IT1	NOR2	-69.69	-85.26	-54.13	<0.001
	IT4	NOR2	-54.03	-69.60	-38.46	<0.001
	NOR13	NOR2	-50.89	-66.46	-35.32	<0.001
D-Arctic	IT1	NOR2	-98.54	-125.05	-72.04	<0.001
	IT4	NOR2	-83.95	-108.47	-59.44	<0.001
	NOR13	NOR2	-99.46	-123.30	-75.62	<0.001
D-Mid-latitude	IT1	NOR2	-102.92	-126.60	-79.24	<0.001
	IT4	NOR2	-90.74	-113.62	-67.87	<0.001
	NOR13	NOR2	-93.46	-115.69	-71.23	<0.001

JA-Ile

Treatment	Accession_1	Accession_2	meandiff	lower	upper	p-adj
D-Arctic	IT1	IT4	38.59	16.69	60.49	<0.001
	IT4	NOR13	-23.06	-45.86	-0.27	0.044

OH-JA

Treatment	Accession_1	Accession_2	meandiff	lower	upper	p-adj
Arctic	IT1	IT4	-908.11	-1580.91	-235.32	0.001
	IT1	NOR2	2232.83	1491.73	2973.92	<0.001
	IT4	NOR2	3140.94	2412.16	3869.71	<0.001
	NOR13	NOR2	2792.38	2063.60	3521.15	<0.001
Mid-latitude	IT1	IT4	-1011.58	-1670.79	-352.38	<0.001
	IT1	NOR13	-1039.16	-1698.36	-379.96	<0.001
	IT1	NOR2	1588.12	867.08	2309.17	<0.001
	IT4	NOR2	2599.71	1878.66	3320.75	<0.001
	NOR13	NOR2	2627.28	1906.24	3348.33	<0.001
D-Arctic	IT4	NOR2	1705.02	332.78	3077.26	0.002
D-Mid-latitude	IT1	NOR2	1761.33	679.59	2843.08	<0.001
	IT4	NOR2	2288.82	1251.51	3326.14	<0.001
	NOR13	NOR2	2394.27	1356.95	3431.58	<0.001

SA

Treatment	Accession_1	Accession_2	meandiff	lower	upper	p-adj
Arctic	IT1	IT4	12280.77	9266.34	15295.21	<0.001
	IT1	NOR13	6038.85	3024.41	9053.29	<0.001
	IT4	NOR13	-6241.92	-9236.72	-3247.12	<0.001
	IT4	NOR2	-9358.20	-12437.40	-6279.03	<0.001
	NOR13	NOR2	-3116.28	-6195.46	-37.11	0.044
Mid-latitude	IT1	IT4	13589.55	10594.75	16584.34	<0.001
	IT1	NOR13	5621.63	2626.83	8616.43	<0.001
	IT1	NOR2	5289.39	2107.23	8471.54	<0.001

	IT4	NOR13	-7967.91	-10962.70	-4973.11	<0.001
	IT4	NOR2	-8300.16	-11482.30	-5118.01	<0.001
D-Arctic	IT1	IT4	16355.03	11731.64	20978.41	<0.001
	IT1	NOR13	9186.58	4563.20	13809.97	<0.001
	IT4	NOR13	-7168.44	-11576.70	-2760.22	<0.001
	IT4	NOR2	-11794.70	-16608.20	-6981.22	<0.001
D-Mid-latitude	IT1	IT4	14144.82	9600.93	18688.72	<0.001
	IT1	NOR13	5869.84	1325.95	10413.74	0.001
	IT4	NOR13	-8274.98	-12683.20	-3866.75	<0.001
	IT4	NOR2	-10431.60	-14839.80	-6023.37	<0.001

Supplementary Table 5. Details of analysis of phytohormones by LC-MS/MS [HPLC 1260 (Agilent Technologies)-QTRAP6500 (SCIEX)] in negative ionisation mode.

**Q1**	**Q3**	**RT (min)**	**Compound**	**Internal std**	**RF**	**DP**	**EP**	**CE**	**CXP**
136.93	93	3.3	SA	D4-SA	1.0	-20	-8	-24	-7
263	153.2	3.4	ABA	D6-ABA	1.0	-20	-12	-22	-2
209.07	59	3.6	JA	D6-JA+D5-JA	1.0	-20	-9	-24	-2
322.19	130.1	3.9	JA-Ile	D6-JA-Ile+D5-JA-Ile	1.0	-50	-4.5	-30	-4
290.9	165.1	4.6	OPDA	D6-JA+D5-JA	1.0	-20	-12	-24	-2
140.93	97	3.3	D4-SA			-20	-8	-24	-7
269	159.2	3.4	D6-ABA			-20	-12	-22	-2
215	59	3.6	D6-JA			-20	-9	-24	-2
214	59	3.6	D5-JA			-20	-9	-24	-2
328.19	130.1	3.9	D6-JA-Ile			-50	-4.5	-30	-4
327.19	130.1	3.9	D5-JA-Ile			-50	-4.5	-30	-4

Supplementary Figure 1. Average phytohormone levels within each treatment over a 24 h period.

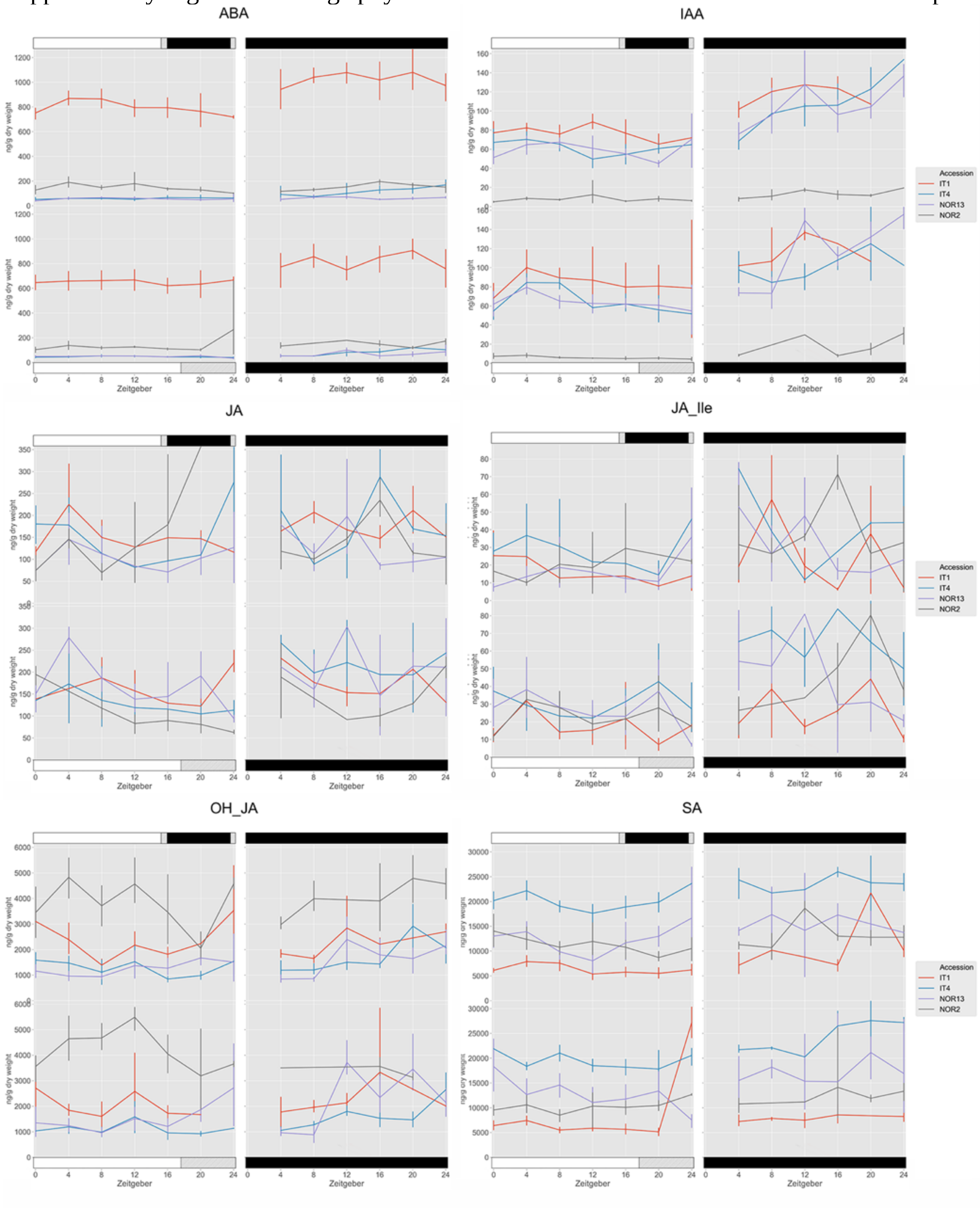

Supplement: Supplementary file 1 — Additional file 1: Supplementary Table 1. List of genes, primers, source, and efficiency values (E%) used in this study. Supplementary Table 2. Significant differences between treatments within each accession. Supplementary Table 3. Significant differences within accessions within each treatment. Supplementary Table 4. Significant differences in phytohormone data between accessions within treatments. Supplementary Table 5. Details of analysis of phytohormones by LC-MS/MS [HPLC 1260 (Agilent Technologies)-QTRAP6500 (SCIEX)] in negative ionisation mode. Supplementary Figure 1. Average phytohormone levels within each treatment over a 24 h period. [file 12870_2023_4491_MOESM1_ESM.pdf]
